# Ep­oxy­cytochalasin H methanol solvate

**DOI:** 10.1107/S1600536810029600

**Published:** 2010-07-31

**Authors:** Li-mei Li, Yang Liu, Tai Yang, Kai-bei Yu, Qiang Zou

**Affiliations:** a Scientific Research Center, Chengdu Medical College, Chengdu 610083, People’s Republic of China; b Chengdu Institute of Organic Chemistry, Chinese Academy of Sciences, Chengdu 610041, People’s Republic of China

## Abstract

In the title solvate, C_30_H_39_NO_5_·CH_4_O {systematic name: 21-acet­oxy-18,21-dihy­droxy-5,6,16,18-tetra­methyl-10-phenyl-6,7-ep­oxy-[11]cytochalasa-13,19-dien-1-one methanol solvate}, the organic mol­ecule exhibits the tetra­cyclic terpenoid skeleton of cytochalasin, consisting of fused five-, six-, three- and 11-membered rings. The five-membered ring adopts an envelope conformation, while the six-membered ring is in a boat conformation. The ep­oxy O atom on the six-membered ring is pointing away from the five-membered ring. An inter­stitial methanol solvent mol­ecule is hydrogen bonded to the cytochalasin mol­ecules and inter­molecular O—H⋯O and N—H⋯O hydrogen bonds connect the mol­ecules into infinite chains along the (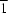
10) direction.

## Related literature

For the isolation and structure elucidation of the title compound, see: Cole *et al.* (1982 ▶). For related structures, see: Buchi *et al.* (1973 ▶); Beno *et al.* (1977 ▶); Capasso *et al.* (1988 ▶); Edwards & Maitland *et al.* (1989 ▶); Chen *et al.* (1993 ▶); Feng *et al.* (2002 ▶); Evidente *et al.* (2002 ▶, 2003 ▶); Rochfort *et al.* (2008 ▶); Herath *et al.* (2005 ▶); Ding *et al.* (2006 ▶). For total syntheses of related compounds, see: Haidle & Myers *et al.* (2004 ▶). For the biological activity of related compounds, see: Hirose *et al.* (1990 ▶); Lingham *et al.* (1992 ▶); Burres *et al.* (1992 ▶); Meurer-Grimes *et al.* (2005 ▶)
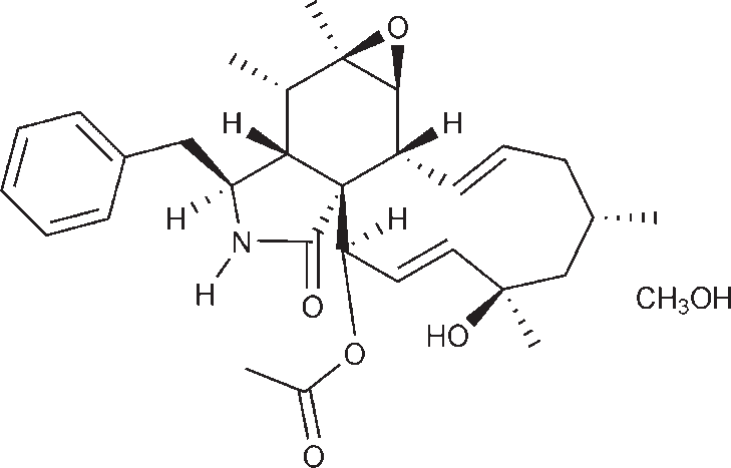


## Experimental

### 

#### Crystal data

C_30_H_39_NO_5_·CH_4_O
*M* *_r_* = 525.66
Triclinic, 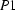

*a* = 8.367 (2) Å
*b* = 8.5937 (18) Å
*c* = 10.917 (3) Å
α = 75.312 (9)°
β = 87.779 (10)°
γ = 68.150 (8)°
*V* = 703.4 (3) Å^3^
*Z* = 1
Mo *K*α radiation
μ = 0.09 mm^−1^
*T* = 93 K
0.50 × 0.50 × 0.28 mm

#### Data collection

Rigaku SPIDER diffractometer
7075 measured reflections
3175 independent reflections
3015 reflections with *I* > 2σ(*I*)
*R*_int_ = 0.022

#### Refinement

*R*[*F* ^2^ > 2σ(*F* ^2^)] = 0.030
*wR*(*F* ^2^) = 0.070
*S* = 1.00
3175 reflections
361 parameters
3 restraints
H atoms treated by a mixture of independent and constrained refinement
Δρ_max_ = 0.23 e Å^−3^
Δρ_min_ = −0.16 e Å^−3^

### 

Data collection: *RAPID-AUTO* (Rigaku, 2004 ▶); cell refinement: *RAPID-AUTO*; data reduction: *RAPID-AUTO*; program(s) used to solve structure: *SHELXS97* (Sheldrick, 2008 ▶); program(s) used to refine structure: *SHELXL97* (Sheldrick, 2008 ▶); molecular graphics: *ORTEP-3 for Windows* (Farrugia, 1997 ▶); software used to prepare material for publication: *SHELXL97*.

## Supplementary Material

Crystal structure: contains datablocks global, I. DOI: 10.1107/S1600536810029600/zl2284sup1.cif

Structure factors: contains datablocks I. DOI: 10.1107/S1600536810029600/zl2284Isup2.hkl

Additional supplementary materials:  crystallographic information; 3D view; checkCIF report

## Figures and Tables

**Table 1:** Hydrogen-bond geometry (Å, °)

*D*—H⋯*A*	*D*—H	H⋯*A*	*D*⋯*A*	*D*—H⋯*A*
O3—H3*O*⋯O6	0.83 (3)	1.94 (3)	2.722 (2)	157 (3)
N2—H2*N*⋯O3^i^	0.90 (3)	1.97 (3)	2.867 (2)	175 (2)
O6—H6*O*⋯O2^ii^	0.84 (3)	1.90 (3)	2.736 (2)	176 (3)

Symmetry codes: (i) 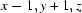; (ii) 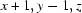.

**Fig. 1.**
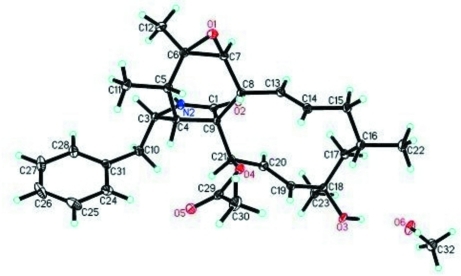
View of the title molecule showing the atom-numbering scheme. Displacement ellipsoids are drawn at the 50% probability level.

**Fig. 2.**
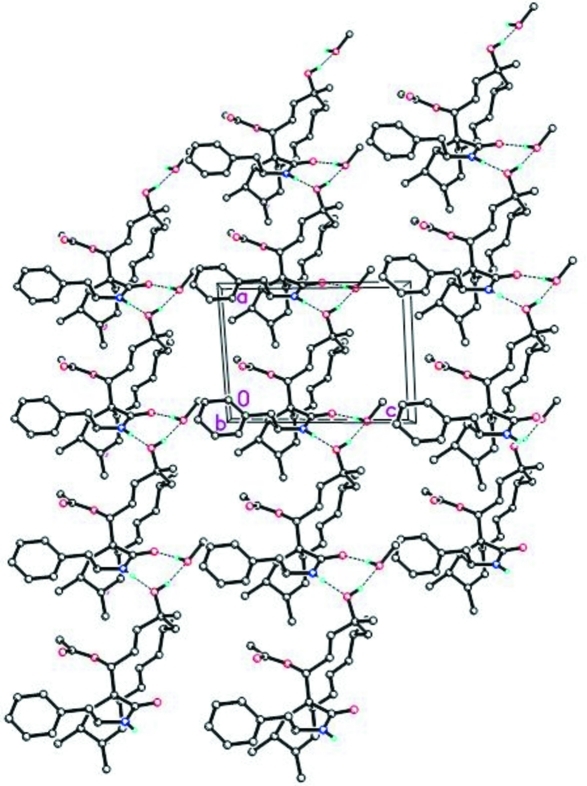
The crystal packing of the title molecule, viewed down the a axis. Most of the H-atoms were omitted for clarity. The intermolecular O—H···O and N—H···O hydrogen bonds are marked as dashed lines.

## supplementary crystallographic information

### Comment

The fungus *Phomopsis sp.108* is a plant pathogen. Cytochalasins from this fungus inhibit a variety of cellular movements, including cell division and motility, and cause changes in cell shape. In our study on the chemical constituents of the secondary metabolites from this fungus, the title compound was isolated. Its structure was elucidated by spectroscopic analysis and was confirmed by single-crystal X-ray diffraction analysis. The asymmetric unit (Fig. 1) of the title compound contains a cytochalasin-structure and a methanol molecule. The cytochalasin-structure has a tetracyclic terpenoid skeleton, consisting of fused five-, six-, three- and eleven-membered rings (A: C1/N2/C3—C4/C9, B: C4—C9, C: C6—C7/O1, D: C9—C8/C13—C21). *Cis* junctions are present between ring A and ring B and also ring B and ring C, while there is a *trans* junction is between ring B and ring D. Ring A adopts an envelope conformation while ring B is in a chair conformation. The epoxy atom in ring R is pointing away from ring A. Intermolecular O—H···O and N—H···O hydrogen bonds are present in the crystal structure, and an eight-membered ring is formed by the hydrogen bonding interaction between two cytochalasin molecules and one methanol molecule (Fig. 2).

### Experimental

A solid-state fermented rice culture (4 kg) of *Phomopsis sp.108* was extracted with ethyl acetate. The solvent was evaporated *in vacuo* to afford a residue (60.0 g). This residue was separated over a silica gel column (Silica gel: 800 g) eluted with petroleum ether/acetone (3:1, 4000 ml) to afford fractions A (18.0 g), B (6.5 g), C (4.8 g), D (5.5 g), E (7.6 g). The title compound (0.5 g) was deposited repeatedly from fraction C in the solvent system of petroleum ether/acetone (2:1) (The *Rf* value of the title compound in this solvent system was 0.35). It was then crystallized in methanol by slow evaporation in a shady place to afford suitable crystals for single-crystal X-ray diffraction analysis.

^13^C NMR (150 MHz, CDCl_3_, δ, p.p.m.): 175.0(C1), 170.0(C29), 138.0(C14), 137.2(C31), 136.0(C20), 130.2(C24, 28), 128.7(C25, 27), 127.6(C19), 127.1(C13), 125.5(C26), 76.0(C21), 74.1(C18), 62.9(C7), 57.0(C6), 53.8(C3), 53.7(C17), 53.5(C9), 51.1(C4), 45.7(C15), 45.0(C8), 43.1(C10), 36.6(C5), 30.1(C16), 28.6(C23), 26.4(C22), 20.6(C30), 19.5(C12), 13.0(C11). The ^13^C NMR values are in accordance with those reported by Cole (Cole *et al.* (1982)).

### Refinement

All hydrogen atoms were located geometrically with C—H distances of 0.95–1.00 Å. The H of O-3, O-6 and N-2 were located in difference Fourier maps and were refined with O—H distances of 0.83 (3)–0.84 (3) Å. H atoms on carbon atoms were placed geometrically with C—H distances of 0.95–1.00 Å (*U*_iso_(H) = 1.2*U*_eq_(C)). The absolute configuration could not be determined from the X-ray analysis, owing to the absence of significant anomalous scatterers, and Friedel pairs were merged. However, when the title compound was orginally isolated by Cole (Cole *et al.* (1982)), its absolute configuration was confirmed by comparison with a similar compound (Cytochalasin H) of which the absolute configuration was previously reported by Beno (Beno *et al.* (1977)). Thus the absolute configuration of the title compound was assigned by a comparison between the measured optical rotatory power ([α]^25^_D_=-91° (c=0.1, CHCl_3_)) and the value ([α]^30^_D_=-84.68° (c=0.33, CHCl_3_)) reported by Cole (Cole *et al.* (1982)).

### Crystal data

**Table:**

C_30_H_39_NO_5_·CH_4_O	*F*(000) = 284
*M**_r_* = 525.66	*D*_x_ = 1.241 Mg m^−^^3^
Triclinic, *P*1	Melting point: 398 K
*a* = 8.367 (2) Å	Mo *K*α radiation, λ = 0.71073 Å
*b* = 8.5937 (18) Å	Cell parameters from 2524 reflections
*c* = 10.917 (3) Å	θ = 3.2–27.5°
α = 75.312 (9)°	µ = 0.09 mm^−^^1^
β = 87.779 (10)°	*T* = 93 K
γ = 68.150 (8)°	Block, colorless
*V* = 703.4 (3) Å^3^	0.50 × 0.50 × 0.28 mm
*Z* = 1	

### Data collection

**Table:**

Rigaku SPIDER diffractometer	3015 reflections with *I* > 2σ(*I*)
Radiation source: Rotating Anode	*R*_int_ = 0.022
graphite	θ_max_ = 27.5°, θ_min_ = 3.2°
ω scans	*h* = −10→10
7075 measured reflections	*k* = −11→10
3175 independent reflections	*l* = −14→14

### Refinement

**Table:**

Refinement on *F*^2^	Primary atom site location: structure-invariant direct methods
Least-squares matrix: full	Secondary atom site location: difference Fourier map
*R*[*F*^2^ > 2σ(*F*^2^)] = 0.030	Hydrogen site location: inferred from neighbouring sites
*wR*(*F*^2^) = 0.070	H atoms treated by a mixture of independent and constrained refinement
*S* = 1.00	*w* = 1/[σ^2^(*F*_o_^2^) + (0.0338*P*)^2^ + 0.116*P*] where *P* = (*F*_o_^2^ + 2*F*_c_^2^)/3
3175 reflections	(Δ/σ)_max_ < 0.001
361 parameters	Δρ_max_ = 0.23 e Å^−^^3^
3 restraints	Δρ_min_ = −0.16 e Å^−^^3^

### Special details

**Table:**

Experimental. ^13^C NMR (150 MHz, CDCl_3_, δ, p.p.m.): 175.0(C1), 170.0(C29), 138.0(C14), 137.2(C31), 136.0(C20), 130.2(C24,28), 128.7(C25, 27), 127.6(C19), 127.1(C13), 125.5(C26), 76.0(C21), 74.1(C18), 62.9(C7), 57.0(C6), 53.8(C3), 53.7(C17), 53.5(C9), 51.1(C4), 45.7(C15), 45.0(C8), 43.1(C10), 36.6(C5), 30.1(C16), 28.6(C23), 26.4(C22), 20.6(C30), 19.5(C12), 13.0(C11). And they are in accordance with those reported by Cole (Cole *et al.* (1982)).
Geometry. All e.s.d.'s (except the e.s.d. in the dihedral angle between two l.s. planes) are estimated using the full covariance matrix. The cell e.s.d.'s are taken into account individually in the estimation of e.s.d.'s in distances, angles and torsion angles; correlations between e.s.d.'s in cell parameters are only used when they are defined by crystal symmetry. An approximate (isotropic) treatment of cell e.s.d.'s is used for estimating e.s.d.'s involving l.s. planes.
Refinement. Refinement of *F*^2^ against ALL reflections. The weighted *R*-factor *wR* and goodness of fit *S* are based on *F*^2^, conventional *R*-factors *R* are based on *F*, with *F* set to zero for negative *F*^2^. The threshold expression of *F*^2^ > σ(*F*^2^) is used only for calculating *R*-factors(gt) *etc*. and is not relevant to the choice of reflections for refinement. *R*-factors based on *F*^2^ are statistically about twice as large as those based on *F*, and *R*- factors based on ALL data will be even larger.

### Fractional atomic coordinates and isotropic or equivalent isotropic displacement parameters (Å^2^)

**Table:**

	*x*	*y*	*z*	*U*_iso_*/*U*_eq_	
O1	−0.26599 (18)	0.47464 (18)	0.27188 (13)	0.0196 (3)	
O2	0.05816 (17)	0.57776 (17)	0.56786 (12)	0.0187 (3)	
O3	0.80244 (17)	0.04100 (17)	0.57468 (13)	0.0173 (3)	
O4	0.34749 (16)	0.40110 (17)	0.24838 (12)	0.0161 (3)	
O5	0.40913 (18)	0.60342 (19)	0.09849 (13)	0.0233 (3)	
N2	−0.0535 (2)	0.8235 (2)	0.40622 (15)	0.0155 (3)	
C1	0.0353 (2)	0.6548 (2)	0.45346 (17)	0.0145 (4)	
C3	−0.0527 (2)	0.8866 (2)	0.26910 (17)	0.0141 (4)	
H3	−0.1731	0.9614	0.2336	0.017*	
C4	0.0084 (2)	0.7177 (2)	0.22278 (16)	0.0134 (3)	
H4	0.0976	0.7227	0.1598	0.016*	
C5	−0.1383 (2)	0.6900 (2)	0.15966 (17)	0.0151 (4)	
H5	−0.0812	0.5846	0.1273	0.018*	
C6	−0.2551 (2)	0.6432 (2)	0.26100 (17)	0.0158 (4)	
C7	−0.1616 (2)	0.4928 (2)	0.36551 (17)	0.0165 (4)	
H7	−0.2082	0.4926	0.4513	0.020*	
C8	0.0336 (2)	0.4143 (2)	0.36131 (17)	0.0148 (4)	
H8	0.0586	0.3724	0.2825	0.018*	
C9	0.0983 (2)	0.5674 (2)	0.34475 (17)	0.0134 (3)	
C10	0.0622 (2)	0.9935 (2)	0.23827 (17)	0.0171 (4)	
H10A	0.0325	1.0756	0.2925	0.021*	
H10B	0.1837	0.9139	0.2611	0.021*	
C11	−0.2324 (3)	0.8372 (3)	0.04402 (18)	0.0205 (4)	
H11A	−0.3176	0.8074	0.0058	0.025*	
H11B	−0.1488	0.8540	−0.0183	0.025*	
H11C	−0.2910	0.9444	0.0704	0.025*	
C12	−0.4211 (2)	0.7803 (3)	0.28126 (19)	0.0204 (4)	
H12A	−0.5006	0.8201	0.2063	0.024*	
H12B	−0.3978	0.8785	0.2950	0.024*	
H12C	−0.4732	0.7318	0.3557	0.024*	
C13	0.1132 (2)	0.2589 (2)	0.47220 (18)	0.0168 (4)	
H13	0.0727	0.2658	0.5539	0.020*	
C14	0.2369 (2)	0.1131 (2)	0.46122 (18)	0.0172 (4)	
H14	0.2774	0.1110	0.3789	0.021*	
C15	0.3193 (2)	−0.0482 (2)	0.56493 (18)	0.0186 (4)	
H15A	0.3111	−0.1477	0.5393	0.022*	
H15B	0.2547	−0.0368	0.6421	0.022*	
C16	0.5108 (2)	−0.0869 (2)	0.59701 (18)	0.0183 (4)	
H16	0.5685	−0.0715	0.5153	0.022*	
C17	0.5316 (2)	0.0393 (3)	0.66833 (18)	0.0177 (4)	
H17A	0.4150	0.1189	0.6813	0.021*	
H17B	0.5883	−0.0291	0.7532	0.021*	
C18	0.6351 (2)	0.1496 (2)	0.60366 (17)	0.0151 (4)	
C19	0.5449 (2)	0.2644 (2)	0.47624 (17)	0.0152 (4)	
H19	0.5953	0.2345	0.4018	0.018*	
C20	0.4017 (2)	0.4024 (3)	0.46214 (17)	0.0169 (4)	
H20	0.3600	0.4394	0.5362	0.020*	
C21	0.2980 (2)	0.5068 (2)	0.33880 (17)	0.0143 (4)	
H21	0.3281	0.6116	0.3058	0.017*	
C22	0.5994 (3)	−0.2758 (3)	0.6732 (2)	0.0287 (5)	
H22A	0.5433	−0.2951	0.7530	0.034*	
H22B	0.7213	−0.3001	0.6915	0.034*	
H22C	0.5903	−0.3531	0.6238	0.034*	
C23	0.6591 (3)	0.2519 (3)	0.69262 (18)	0.0193 (4)	
H23A	0.7233	0.1710	0.7710	0.023*	
H23B	0.5459	0.3267	0.7127	0.023*	
H23C	0.7235	0.3235	0.6512	0.023*	
C24	0.1621 (3)	1.0297 (3)	0.0148 (2)	0.0231 (4)	
H24	0.2495	0.9170	0.0403	0.028*	
C25	0.1483 (3)	1.1291 (3)	−0.1103 (2)	0.0333 (5)	
H25	0.2264	1.0832	−0.1693	0.040*	
C26	0.0226 (4)	1.2931 (3)	−0.1488 (2)	0.0346 (6)	
H26	0.0144	1.3602	−0.2338	0.042*	
C27	−0.0913 (4)	1.3590 (3)	−0.0630 (2)	0.0331 (5)	
H27	−0.1783	1.4719	−0.0890	0.040*	
C28	−0.0791 (3)	1.2605 (3)	0.06136 (19)	0.0235 (4)	
H28	−0.1586	1.3066	0.1196	0.028*	
C29	0.4050 (2)	0.4611 (3)	0.13584 (17)	0.0180 (4)	
C30	0.4606 (3)	0.3225 (3)	0.0651 (2)	0.0293 (5)	
H30A	0.5033	0.3652	−0.0163	0.035*	
H30B	0.3620	0.2928	0.0500	0.035*	
H30C	0.5526	0.2191	0.1155	0.035*	
C31	0.0484 (2)	1.0952 (2)	0.10157 (18)	0.0165 (4)	
O6	1.0050 (2)	−0.2459 (2)	0.75153 (14)	0.0272 (3)	
C32	1.1481 (3)	−0.3222 (3)	0.8413 (2)	0.0270 (5)	
H32A	1.1523	−0.2359	0.8837	0.032*	
H32B	1.2546	−0.3629	0.7979	0.032*	
H32C	1.1363	−0.4205	0.9044	0.032*	
H6O	1.023 (4)	−0.304 (4)	0.698 (3)	0.036 (8)*	
H3O	0.856 (4)	−0.027 (4)	0.641 (3)	0.043 (8)*	
H2N	−0.098 (3)	0.897 (3)	0.455 (2)	0.025 (6)*	

### Atomic displacement parameters (Å^2^)

**Table:**

	*U*^11^	*U*^22^	*U*^33^	*U*^12^	*U*^13^	*U*^23^
O1	0.0189 (7)	0.0198 (7)	0.0219 (7)	−0.0094 (6)	−0.0008 (5)	−0.0053 (6)
O2	0.0231 (7)	0.0167 (7)	0.0131 (6)	−0.0052 (6)	0.0016 (5)	−0.0020 (5)
O3	0.0116 (6)	0.0165 (7)	0.0176 (7)	0.0010 (5)	−0.0018 (5)	−0.0032 (6)
O4	0.0153 (6)	0.0157 (7)	0.0150 (6)	−0.0027 (5)	0.0010 (5)	−0.0050 (5)
O5	0.0200 (7)	0.0246 (8)	0.0202 (7)	−0.0062 (6)	0.0026 (5)	−0.0004 (6)
N2	0.0172 (8)	0.0126 (8)	0.0142 (8)	−0.0023 (6)	0.0018 (6)	−0.0041 (6)
C1	0.0118 (8)	0.0153 (9)	0.0161 (9)	−0.0048 (7)	0.0012 (6)	−0.0042 (7)
C3	0.0139 (8)	0.0129 (9)	0.0129 (8)	−0.0027 (7)	0.0003 (6)	−0.0024 (7)
C4	0.0123 (8)	0.0134 (9)	0.0118 (8)	−0.0025 (7)	0.0003 (6)	−0.0023 (7)
C5	0.0143 (9)	0.0154 (9)	0.0139 (8)	−0.0038 (7)	−0.0006 (6)	−0.0035 (7)
C6	0.0137 (9)	0.0170 (10)	0.0179 (9)	−0.0062 (7)	−0.0011 (7)	−0.0055 (7)
C7	0.0153 (9)	0.0188 (10)	0.0168 (9)	−0.0084 (8)	0.0016 (7)	−0.0042 (7)
C8	0.0150 (9)	0.0136 (9)	0.0153 (9)	−0.0050 (7)	0.0005 (7)	−0.0034 (7)
C9	0.0130 (8)	0.0115 (9)	0.0134 (8)	−0.0027 (7)	−0.0001 (6)	−0.0017 (7)
C10	0.0192 (9)	0.0161 (10)	0.0165 (9)	−0.0073 (8)	−0.0021 (7)	−0.0034 (7)
C11	0.0208 (10)	0.0214 (11)	0.0162 (9)	−0.0049 (8)	−0.0036 (7)	−0.0037 (8)
C12	0.0147 (9)	0.0244 (11)	0.0220 (9)	−0.0064 (8)	0.0012 (7)	−0.0076 (8)
C13	0.0179 (9)	0.0166 (9)	0.0169 (9)	−0.0082 (8)	0.0002 (7)	−0.0029 (7)
C14	0.0193 (9)	0.0165 (10)	0.0171 (9)	−0.0096 (8)	−0.0019 (7)	−0.0018 (7)
C15	0.0191 (9)	0.0135 (10)	0.0230 (10)	−0.0066 (8)	−0.0017 (7)	−0.0030 (8)
C16	0.0165 (9)	0.0143 (9)	0.0221 (10)	−0.0041 (7)	−0.0015 (7)	−0.0033 (8)
C17	0.0179 (9)	0.0177 (10)	0.0162 (9)	−0.0066 (8)	0.0007 (7)	−0.0025 (7)
C18	0.0126 (8)	0.0142 (9)	0.0155 (9)	−0.0021 (7)	−0.0001 (6)	−0.0028 (7)
C19	0.0155 (9)	0.0154 (9)	0.0151 (9)	−0.0062 (7)	0.0020 (7)	−0.0041 (7)
C20	0.0164 (9)	0.0194 (10)	0.0145 (9)	−0.0053 (8)	0.0002 (7)	−0.0056 (7)
C21	0.0156 (8)	0.0121 (9)	0.0144 (8)	−0.0041 (7)	0.0017 (7)	−0.0038 (7)
C22	0.0228 (11)	0.0158 (10)	0.0417 (13)	−0.0042 (9)	−0.0060 (9)	−0.0009 (9)
C23	0.0213 (10)	0.0164 (10)	0.0192 (9)	−0.0054 (8)	−0.0020 (7)	−0.0048 (8)
C24	0.0233 (10)	0.0235 (11)	0.0259 (10)	−0.0114 (9)	0.0062 (8)	−0.0090 (9)
C25	0.0455 (14)	0.0428 (15)	0.0246 (11)	−0.0288 (12)	0.0159 (10)	−0.0140 (11)
C26	0.0605 (17)	0.0341 (13)	0.0178 (10)	−0.0319 (13)	0.0010 (10)	0.0006 (9)
C27	0.0493 (15)	0.0190 (11)	0.0265 (11)	−0.0121 (11)	−0.0105 (10)	0.0024 (9)
C28	0.0305 (11)	0.0186 (10)	0.0201 (10)	−0.0081 (9)	0.0004 (8)	−0.0040 (8)
C29	0.0129 (8)	0.0209 (10)	0.0144 (9)	−0.0011 (7)	−0.0004 (7)	−0.0027 (7)
C30	0.0300 (12)	0.0277 (12)	0.0203 (10)	0.0016 (9)	0.0032 (8)	−0.0086 (9)
C31	0.0197 (9)	0.0150 (9)	0.0185 (9)	−0.0107 (8)	0.0000 (7)	−0.0040 (7)
O6	0.0292 (8)	0.0218 (8)	0.0222 (8)	0.0026 (6)	−0.0077 (6)	−0.0086 (6)
C32	0.0262 (11)	0.0287 (12)	0.0241 (10)	−0.0094 (9)	−0.0045 (8)	−0.0037 (9)

### Geometric parameters (Å, °)

**Table:**

O1—C7	1.444 (2)	C15—C16	1.545 (3)
O1—C6	1.460 (2)	C15—H15A	0.9900
O2—C1	1.241 (2)	C15—H15B	0.9900
O3—C18	1.440 (2)	C16—C22	1.531 (3)
O3—H3O	0.83 (3)	C16—C17	1.547 (3)
O4—C29	1.351 (2)	C16—H16	1.0000
O4—C21	1.454 (2)	C17—C18	1.540 (3)
O5—C29	1.200 (3)	C17—H17A	0.9900
N2—C1	1.330 (2)	C17—H17B	0.9900
N2—C3	1.458 (2)	C18—C19	1.519 (3)
N2—H2N	0.90 (3)	C18—C23	1.528 (3)
C1—C9	1.536 (2)	C19—C20	1.315 (3)
C3—C10	1.535 (3)	C19—H19	0.9500
C3—C4	1.557 (2)	C20—C21	1.506 (3)
C3—H3	1.0000	C20—H20	0.9500
C4—C5	1.551 (2)	C21—H21	1.0000
C4—C9	1.578 (2)	C22—H22A	0.9800
C4—H4	1.0000	C22—H22B	0.9800
C5—C6	1.524 (2)	C22—H22C	0.9800
C5—C11	1.530 (3)	C23—H23A	0.9800
C5—H5	1.0000	C23—H23B	0.9800
C6—C7	1.467 (3)	C23—H23C	0.9800
C6—C12	1.502 (3)	C24—C31	1.386 (3)
C7—C8	1.521 (3)	C24—C25	1.398 (3)
C7—H7	1.0000	C24—H24	0.9500
C8—C13	1.508 (3)	C25—C26	1.378 (4)
C8—C9	1.569 (2)	C25—H25	0.9500
C8—H8	1.0000	C26—C27	1.379 (4)
C9—C21	1.559 (2)	C26—H26	0.9500
C10—C31	1.510 (3)	C27—C28	1.390 (3)
C10—H10A	0.9900	C27—H27	0.9500
C10—H10B	0.9900	C28—C31	1.393 (3)
C11—H11A	0.9800	C28—H28	0.9500
C11—H11B	0.9800	C29—C30	1.502 (3)
C11—H11C	0.9800	C30—H30A	0.9800
C12—H12A	0.9800	C30—H30B	0.9800
C12—H12B	0.9800	C30—H30C	0.9800
C12—H12C	0.9800	O6—C32	1.416 (3)
C13—C14	1.325 (3)	O6—H6O	0.84 (3)
C13—H13	0.9500	C32—H32A	0.9800
C14—C15	1.494 (3)	C32—H32B	0.9800
C14—H14	0.9500	C32—H32C	0.9800
			
C7—O1—C6	60.70 (12)	C16—C15—H15B	108.9
C18—O3—H3O	109 (2)	H15A—C15—H15B	107.7
C29—O4—C21	119.78 (14)	C22—C16—C15	109.93 (16)
C1—N2—C3	115.28 (15)	C22—C16—C17	111.15 (17)
C1—N2—H2N	122.7 (16)	C15—C16—C17	112.02 (15)
C3—N2—H2N	121.5 (16)	C22—C16—H16	107.9
O2—C1—N2	125.31 (17)	C15—C16—H16	107.9
O2—C1—C9	124.87 (17)	C17—C16—H16	107.9
N2—C1—C9	109.76 (15)	C18—C17—C16	116.27 (15)
N2—C3—C10	109.65 (14)	C18—C17—H17A	108.2
N2—C3—C4	103.39 (14)	C16—C17—H17A	108.2
C10—C3—C4	115.66 (15)	C18—C17—H17B	108.2
N2—C3—H3	109.3	C16—C17—H17B	108.2
C10—C3—H3	109.3	H17A—C17—H17B	107.4
C4—C3—H3	109.3	O3—C18—C19	105.29 (14)
C5—C4—C3	113.89 (14)	O3—C18—C23	108.79 (15)
C5—C4—C9	112.81 (14)	C19—C18—C23	113.22 (15)
C3—C4—C9	104.64 (14)	O3—C18—C17	110.76 (15)
C5—C4—H4	108.4	C19—C18—C17	109.41 (15)
C3—C4—H4	108.4	C23—C18—C17	109.31 (15)
C9—C4—H4	108.4	C20—C19—C18	124.34 (16)
C6—C5—C11	114.86 (16)	C20—C19—H19	117.8
C6—C5—C4	109.27 (14)	C18—C19—H19	117.8
C11—C5—C4	113.97 (15)	C19—C20—C21	125.68 (17)
C6—C5—H5	106.0	C19—C20—H20	117.2
C11—C5—H5	106.0	C21—C20—H20	117.2
C4—C5—H5	106.0	O4—C21—C20	108.18 (15)
O1—C6—C7	59.09 (11)	O4—C21—C9	107.03 (14)
O1—C6—C12	114.16 (15)	C20—C21—C9	115.88 (15)
C7—C6—C12	120.90 (16)	O4—C21—H21	108.5
O1—C6—C5	114.31 (15)	C20—C21—H21	108.5
C7—C6—C5	113.23 (15)	C9—C21—H21	108.5
C12—C6—C5	120.09 (16)	C16—C22—H22A	109.5
O1—C7—C6	60.21 (12)	C16—C22—H22B	109.5
O1—C7—C8	118.13 (15)	H22A—C22—H22B	109.5
C6—C7—C8	116.79 (15)	C16—C22—H22C	109.5
O1—C7—H7	116.6	H22A—C22—H22C	109.5
C6—C7—H7	116.6	H22B—C22—H22C	109.5
C8—C7—H7	116.6	C18—C23—H23A	109.5
C13—C8—C7	111.45 (15)	C18—C23—H23B	109.5
C13—C8—C9	116.96 (15)	H23A—C23—H23B	109.5
C7—C8—C9	106.34 (14)	C18—C23—H23C	109.5
C13—C8—H8	107.2	H23A—C23—H23C	109.5
C7—C8—H8	107.2	H23B—C23—H23C	109.5
C9—C8—H8	107.2	C31—C24—C25	120.2 (2)
C1—C9—C21	111.33 (14)	C31—C24—H24	119.9
C1—C9—C8	108.67 (14)	C25—C24—H24	119.9
C21—C9—C8	112.47 (14)	C26—C25—C24	120.7 (2)
C1—C9—C4	103.13 (14)	C26—C25—H25	119.7
C21—C9—C4	109.56 (14)	C24—C25—H25	119.7
C8—C9—C4	111.29 (14)	C25—C26—C27	119.5 (2)
C31—C10—C3	115.31 (15)	C25—C26—H26	120.2
C31—C10—H10A	108.4	C27—C26—H26	120.2
C3—C10—H10A	108.4	C26—C27—C28	120.2 (2)
C31—C10—H10B	108.4	C26—C27—H27	119.9
C3—C10—H10B	108.4	C28—C27—H27	119.9
H10A—C10—H10B	107.5	C27—C28—C31	120.8 (2)
C5—C11—H11A	109.5	C27—C28—H28	119.6
C5—C11—H11B	109.5	C31—C28—H28	119.6
H11A—C11—H11B	109.5	O5—C29—O4	125.02 (18)
C5—C11—H11C	109.5	O5—C29—C30	125.77 (18)
H11A—C11—H11C	109.5	O4—C29—C30	109.20 (17)
H11B—C11—H11C	109.5	C29—C30—H30A	109.5
C6—C12—H12A	109.5	C29—C30—H30B	109.5
C6—C12—H12B	109.5	H30A—C30—H30B	109.5
H12A—C12—H12B	109.5	C29—C30—H30C	109.5
C6—C12—H12C	109.5	H30A—C30—H30C	109.5
H12A—C12—H12C	109.5	H30B—C30—H30C	109.5
H12B—C12—H12C	109.5	C24—C31—C28	118.69 (18)
C14—C13—C8	123.18 (17)	C24—C31—C10	121.66 (18)
C14—C13—H13	118.4	C28—C31—C10	119.63 (17)
C8—C13—H13	118.4	C32—O6—H6O	108 (2)
C13—C14—C15	126.72 (18)	O6—C32—H32A	109.5
C13—C14—H14	116.6	O6—C32—H32B	109.5
C15—C14—H14	116.6	H32A—C32—H32B	109.5
C14—C15—C16	113.25 (15)	O6—C32—H32C	109.5
C14—C15—H15A	108.9	H32A—C32—H32C	109.5
C16—C15—H15A	108.9	H32B—C32—H32C	109.5
C14—C15—H15B	108.9		
			
C3—N2—C1—O2	176.02 (17)	C5—C4—C9—C21	132.78 (15)
C3—N2—C1—C9	−6.7 (2)	C3—C4—C9—C21	−102.89 (16)
C1—N2—C3—C10	−107.04 (18)	C5—C4—C9—C8	7.8 (2)
C1—N2—C3—C4	16.9 (2)	C3—C4—C9—C8	132.09 (15)
N2—C3—C4—C5	104.44 (16)	N2—C3—C10—C31	−168.78 (15)
C10—C3—C4—C5	−135.70 (16)	C4—C3—C10—C31	74.8 (2)
N2—C3—C4—C9	−19.19 (17)	C7—C8—C13—C14	136.71 (19)
C10—C3—C4—C9	100.67 (17)	C9—C8—C13—C14	−100.7 (2)
C3—C4—C5—C6	−71.29 (19)	C8—C13—C14—C15	−178.15 (17)
C9—C4—C5—C6	47.79 (19)	C13—C14—C15—C16	−112.7 (2)
C3—C4—C5—C11	58.7 (2)	C14—C15—C16—C22	−163.36 (17)
C9—C4—C5—C11	177.80 (15)	C14—C15—C16—C17	72.5 (2)
C7—O1—C6—C12	−112.79 (18)	C22—C16—C17—C18	116.22 (19)
C7—O1—C6—C5	103.53 (17)	C15—C16—C17—C18	−120.37 (18)
C11—C5—C6—O1	110.27 (17)	C16—C17—C18—O3	−54.2 (2)
C4—C5—C6—O1	−120.21 (16)	C16—C17—C18—C19	61.4 (2)
C11—C5—C6—C7	175.47 (15)	C16—C17—C18—C23	−174.07 (16)
C4—C5—C6—C7	−55.0 (2)	O3—C18—C19—C20	−167.40 (17)
C11—C5—C6—C12	−31.1 (2)	C23—C18—C19—C20	−48.7 (2)
C4—C5—C6—C12	98.44 (19)	C17—C18—C19—C20	73.5 (2)
C6—O1—C7—C8	−106.43 (18)	C18—C19—C20—C21	−172.52 (17)
C12—C6—C7—O1	101.40 (18)	C29—O4—C21—C20	−124.04 (17)
C5—C6—C7—O1	−105.38 (16)	C29—O4—C21—C9	110.43 (17)
O1—C6—C7—C8	108.64 (17)	C19—C20—C21—O4	18.5 (3)
C12—C6—C7—C8	−149.96 (17)	C19—C20—C21—C9	138.65 (19)
C5—C6—C7—C8	3.3 (2)	C1—C9—C21—O4	170.55 (14)
O1—C7—C8—C13	−109.94 (18)	C8—C9—C21—O4	48.30 (18)
C6—C7—C8—C13	−178.77 (15)	C4—C9—C21—O4	−76.03 (17)
O1—C7—C8—C9	121.54 (16)	C1—C9—C21—C20	49.8 (2)
C6—C7—C8—C9	52.7 (2)	C8—C9—C21—C20	−72.4 (2)
O2—C1—C9—C21	−71.7 (2)	C4—C9—C21—C20	163.22 (15)
N2—C1—C9—C21	110.94 (17)	C31—C24—C25—C26	0.2 (3)
O2—C1—C9—C8	52.7 (2)	C24—C25—C26—C27	−0.4 (4)
N2—C1—C9—C8	−124.65 (16)	C25—C26—C27—C28	0.0 (4)
O2—C1—C9—C4	170.86 (17)	C26—C27—C28—C31	0.5 (3)
N2—C1—C9—C4	−6.46 (18)	C21—O4—C29—O5	−5.2 (3)
C13—C8—C9—C1	−68.96 (19)	C21—O4—C29—C30	175.47 (16)
C7—C8—C9—C1	56.26 (18)	C25—C24—C31—C28	0.3 (3)
C13—C8—C9—C21	54.8 (2)	C25—C24—C31—C10	−178.39 (18)
C7—C8—C9—C21	179.99 (14)	C27—C28—C31—C24	−0.6 (3)
C13—C8—C9—C4	178.15 (15)	C27—C28—C31—C10	178.06 (19)
C7—C8—C9—C4	−56.64 (18)	C3—C10—C31—C24	−95.0 (2)
C5—C4—C9—C1	−108.57 (16)	C3—C10—C31—C28	86.4 (2)
C3—C4—C9—C1	15.75 (17)		

### Hydrogen-bond geometry (Å, °)

**Table:**

*D*—H···*A*	*D*—H	H···*A*	*D*···*A*	*D*—H···*A*
O3—H3O···O6	0.83 (3)	1.94 (3)	2.722 (2)	157 (3)
N2—H2N···O3^i^	0.90 (3)	1.97 (3)	2.867 (2)	175 (2)
O6—H6O···O2^ii^	0.84 (3)	1.90 (3)	2.736 (2)	176 (3)

Symmetry codes: (i) *x*−1, *y*+1, *z*; (ii) *x*+1, *y*−1, *z*.
